# Plasma 24-metabolite Panel Predicts Preclinical Transition to Clinical Stages of Alzheimer’s Disease

**DOI:** 10.3389/fneur.2015.00237

**Published:** 2015-11-12

**Authors:** Massimo S. Fiandaca, Xiaogang Zhong, Amrita K. Cheema, Michael H. Orquiza, Swathi Chidambaram, Ming T. Tan, Carole Roan Gresenz, Kevin T. FitzGerald, Mike A. Nalls, Andrew B. Singleton, Mark Mapstone, Howard J. Federoff

**Affiliations:** ^1^Department of Neurology, University of California Irvine, Irvine, CA, USA; ^2^Department of Neurological Surgery, University of California Irvine, Irvine, CA, USA; ^3^Department of Bioinformatics, Biostatistics and Biomathematics, Georgetown University Medical Center, Washington, DC, USA; ^4^Departments of Oncology and Biochemistry, Georgetown University Medical Center, Washington, DC, USA; ^5^Department of Neuroscience, Georgetown University Medical Center, Washington, DC, USA; ^6^School of Medicine, Georgetown University Medical Center, Washington, DC, USA; ^7^Department of Economics, Sociology and Statistics, RAND Corporation, Arlington, VA, USA; ^8^Pellegrino Center for Clinical Bioethics, Georgetown University Medical Center, Washington, DC, USA; ^9^Laboratory of Neurogenetics, National Institute on Aging, National Institutes of Health, Bethesda, MD, USA

**Keywords:** Alzheimer’s disease, biomarkers, economics, ethics, lipids, metabolomics, risk assessment

## Abstract

We recently documented plasma lipid dysregulation in preclinical late-onset Alzheimer’s disease (LOAD). A 10 plasma lipid panel, predicted phenoconversion and provided 90% sensitivity and 85% specificity in differentiating an at-risk group from those that would remain cognitively intact. Despite these encouraging results, low positive predictive values limit the clinical usefulness of this panel as a screening tool in subjects aged 70–80 years or younger. In this report, we re-examine our metabolomic data, analyzing baseline plasma specimens from our group of phenoconverters (*n* = 28) and a matched set of cognitively normal subjects (*n* = 73), and discover and internally validate a panel of 24 plasma metabolites. The new panel provides a classifier with receiver operating characteristic area under the curve for the discovery and internal validation cohort of 1.0 and 0.995 (95% confidence intervals of 1.0–1.0, and 0.981–1.0), respectively. Twenty-two of the 24 metabolites were significantly dysregulated lipids. While positive and negative predictive values were improved compared to our 10-lipid panel, low positive predictive values provide a reality check on the utility of such biomarkers in this age group (or younger). Through inclusion of additional significantly dysregulated analyte species, our new biomarker panel provides greater accuracy in our cohort but remains limited by predictive power. Unfortunately, the novel metabolite panel alone may not provide improvement in counseling and management of at-risk individuals but may further improve selection of subjects for LOAD secondary prevention trials. We expect that external validation will remain challenging due to our stringent study design, especially compared with more diverse subject cohorts. We do anticipate, however, external validation of reduced plasma lipid species as a predictor of phenoconversion to either prodromal or manifest LOAD.

## Introduction

A major push in neurology and neurological research related to late-onset Alzheimer’s disease (LOAD) in the last 5 years has been to better define the preclinical pathological stages that herald the development of clinically overt disease (1). As it relates to this paper, when we use the term AD, we mean LOAD, the most common clinical form of the disease and featuring a combination of genetic and epigenetic etiologies. In this context, we define preclinical LOAD as the separate stages of pathobiologic development that immediately precede prodromal amnestic mild cognitive impairment (aMCI) and manifest LOAD. We define, therefore, aMCI and LOAD to comprise the clinical stages of AD. Since treatments initiated during the preclinical stages may be more effective due to a more receptive brain substrate, the discovery and validation of biomarkers that define such a preclinical period has gained significant momentum (1). Our current investigative efforts focus on defining a more accurate and predictive set of plasma-based metabolomic biomarkers compared to those from our previous study (2). While the majority of LOAD biomarker studies to date have been carried out via case–control comparisons, our investigations arise from data developed from a 5-year longitudinal observation study. Longitudinal studies allow direct assessment of pathobiology during times of transition, while case–control studies primarily infer these transition events by comparing health to disease. Cerebrospinal fluid (CSF), neuroimaging, and a variety of other blood-based biomarkers have also been proposed via case–control analyses (3) but have not gained favor due to their associated risk, cost, and/or lack of requisite sensitivity and specificity values. There are few longitudinal investigations in the literature that define which neurocognitively intact subjects will progress to either prodromal or manifest LOAD. Our recent plasma lipid biomarker study (2) provided receiver operating characteristic area under the curve (ROC AUC) values of 0.96 and 0.92 with 95% confidence interval of 0.93–0.99 and 0.87–0.98, respectively, in the discovery and internal validation cohorts analyzed. The calculated positive predictive value (PPV), but not the negative predictive value (NPV), estimates remained low due to the low prevalence in this age group, arguing against the use of such a panel as a screening tool in a similarly aged, asymptomatic population. While sensitivity and specificity reflect on accuracy provided by a test, predictive values address the meaning of test results given a particular context (i.e., age-dependent prevalence) (4). The discovery and internal validation metabolomic analyses that were originally advanced, however, provided support to the lipid irregularities previously associated with LOAD (5), and our 5-year longitudinal study design allowed identification of biomarkers that predict the pending phenoconversion to the clinical stages of LOAD. Herein, we describe the discovery and internal validation of an expanded panel of plasma metabolites, from the same baseline asymptomatic subjects previously reported (2). The expanded metabolite panel provides increased sensitivity and specificity and improved predictive values within our cohort. In addition, the specific analytes in the panel further strengthen the links between dysregulated brain and plasma lipid species during the preclinical stages of LOAD. Our expanded biomarker panel, therefore, provides significant potential benefits, as well as burdens that must be considered by individuals and society at large. Such a biomarker panel for preclinical LOAD must initially play a role in selecting subjects for secondary prevention trials and, possibly, monitoring their therapeutic success or failure. Eventually, however, it will be critical that biomarker panels of disease stimulate the development of new or repurposed therapeutics. A diagnostic test without an associated viable treatment option is always limited. Eventually, a highly accurate panel such as proposed might be applicable in a general clinical practice, identifying older adults with a high risk of phenoconversion to the clinical stages of LOAD, and allowing initiation of treatment that could modify the course of disease.

## Materials and Methods

### Participants

**Figure 1:**
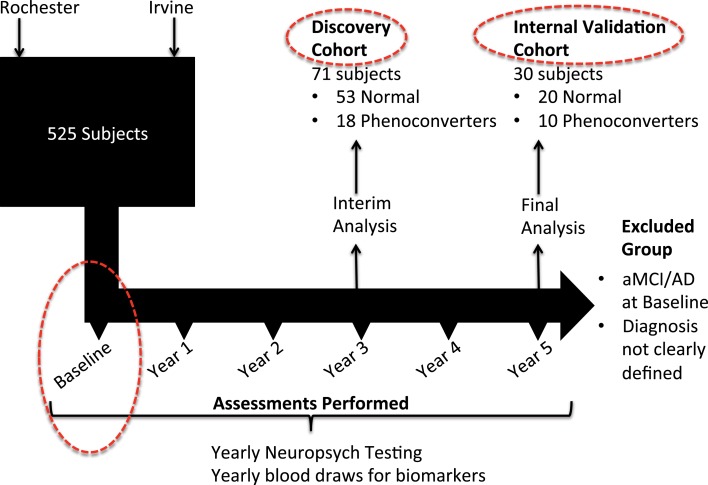
**Schematic representation of overall study design and specific analyses reported in this paper**. Clinical subjects for the 5-year observational study were selected for participation at the University of Rochester and the University of California Irvine. An interim analysis was performed at year 3 of the study, comparing 53 subjects who maintained normal cognition since baseline study entry, to a group of 18 subjects who were cognitively normal at baseline but had phenoconverted to either aMCI or AD by year 3. This group made up our discovery cohort from which initial biomarker discovery was performed. With complete neuropsychological assessments available by study termination, an additional group of 10 subjects were noted to have phenoconverted during year 4 and year 5. This latter group was combined with a group of 20 matched subjects who maintained normal cognition throughout the study, and together were designated as the internal validation group (or cohort). All subjects included in this analysis (Discovery and Internal Validation cohorts) had only their baseline blood specimens assessed for metabolomic biomarker comparisons (dashed red circles).

The study design for this investigation is structured in a manner similar to that used in our original study (2) but features discovery and internal validation sets that include only subjects who maintain a cognitively normal status [normal control (NC)] and those who phenoconvert from cognitive normality at baseline (Converter_pre_) to either aMCI or AD by either year 3 or year 5 of the Rochester/Orange County Aging Study (Figure 1). As part of a 5-year observational study, we enrolled a total of 525 community-dwelling participants from two distinct geographic regions, aged 70 and older, and who were otherwise healthy. Health records and medications were fully documented, and subjects were excluded only if major neurologic or oncologic illness was present. All study participants provided informed consent for study inclusion and use of their neurocognitive results and peripheral blood specimens for analyses. Institutional review boards (IRBs) at each institution approved the protocols and informed consent documents. As opposed to including the incident aMCI/AD group, as described in our original investigation (2), the primary inclusion and comparison for this analysis was limited to those subjects who remained cognitively normal throughout the study and those who phenoconverted to aMCI or AD during the 5-year study. Subjects were continuously enrolled in the study over 5 years. In a planned midpoint analysis, we selected those who remained cognitively normal or phenoconverted from baseline to year 3 for the discovery cohort and those who were subsequently enrolled or who subsequently phenoconverted during year 3 to 5 for the internal validation cohort. As shown in Table 1, the 71 discovery subjects include 53 NC and 18 Converter_pre_ individuals. The discovery cohort Converter_pre_ subjects consisted of 2 individuals who phenoconverted to AD and 16 who transitioned to aMCI. Of this group, three of those converting to aMCI carried an *APOE* ϵ4 allele. The 30 internal validation subjects featured 20 NC and 10 Converter_pre_ individuals. Internal validation cohort phenoconverters consisted of five individuals who developed AD and five meeting criteria for aMCI. In the internal validation cohort, two of the AD converters carried an *APOE* ϵ4 allele. The discovery and internal validation cohorts did not share any common subjects. Figure 1 further depicts how the Converter_pre_ subjects were selected (number that phenoconverted by year 3 and the remaining that phenoconverted by year 5) and matched to NC subjects, for this manuscript as well as our previous lipidomic study. The number of subjects in our discovery (*n* = 71) and internal validation (*n* = 30) groups (or cohorts), therefore, approaches the accepted biostatistical standards (6) for discovery and validation groupings of 2/3 and 1/3, respectively. This study focused solely on biomarker comparisons between subject groups categorized as fulfilling the cognitively normal state (Converter_pre_ vs. NC) at baseline. Excluded from this and our previous analysis (2) were a significant number of the total longitudinal study participants who could not be categorized based on the strict neurocognitive grouping parameters. We believe that rigorous clinical classification is necessary to increase signal in the biological samples for new metabolomic discovery. In any study with clinical characterization such as ours, we can clearly identify the cases (aMCI or LOAD), but not all remaining subjects should be considered NCs. Thus, in our work, we specifically define criteria for NCs and those who do not meet either definition (case or control) are not included in the specific study analysis. Subject data from the excluded individuals are undergoing separate analyses, not specifically related to the diagnosis of LOAD. The goal of this analysis, therefore, was to develop a biomarker model that would more accurately predict whether phenoconversion would or would not occur in cognitively normal subjects of our aging cohort within 5 years from study entry. Herein, we compare those cognitively normal (Converter_pre_ or preclinical LOAD, *n* = 28) individuals, who developed memory impairment, with or without functional impairment, within 5 years of study entry, to those subjects who remained cognitively normal (NC, *n* = 73) over the same 5-year study period (Table 1) (total study group analyzed, *n* = 101). Of the 28 subjects who phenoconverted, 21 developed aMCI, and 7 developed AD within the 5-year study. We reiterate that the 101 subjects in this analysis are a subset of those reported in our previous publication (that also included those with incident aMCI/AD) (2).

**Table 1:** **Discovery and internal validation cohort demographic details**.

Clinical groups	n (M/F)	Mean age years [SD]	Mean education years [SD]	% *APOE* ϵ4
Normal control (NC)				
Discovery	53 (18/35)	81.6 [3.6]	15.7 [2.3]	24.6
Internal validation	20 (9/11)	81.4 [3.3]	15.1 [2.5]	20
Converter_pre_				
Discovery	18 (8/10)	80.7 [2.3]	15.3 [3.1]	16.7
Internal validation	10 (4/6)	79.3 [5.5]	14.5 [1.8]	20
Total discovery	71 (26/45)	81.8 [3.0]	15.5 [2.7]	20.7
Total internal validation	30 (13/17)	80.9 [4.4]	14.8 [2.2]	20.0

*n, number of subjects; F, female subjects; M, male subjects; SD, standard deviation; % *APOE* ϵ4, percent having at least a single *APOE* ϵ4 allele*.

*Gender, age, education, and *APOE* ϵ4 status were not significantly different (Chi-square *p* > 0.05) between discovery and internal validation groups*.

Our discovery and internal validation groups of cognitively normal individuals at baseline assessment (including both NC and Converter_pre_) were matched for age, gender, and education and featured similar *APOE* allele status (Table 1). Our internal validation group consisted of approximately one-third of all subjects included in our analysis and was composed of phenoconverters from years 3 to 5 and their matched set of control subjects. All study participants underwent phlebotomy between 8:00 a.m. and 10:00 a.m., on a yearly basis, while fasting and withholding their morning medications, and as close as possible to the same day each year of study participation. Blood specimens were initially placed on ice, and the blood components were separated within 24 h, yielding multiple plasma aliquots that were frozen immediately thereafter at −80°C until undergoing metabolomic analyses. Smaller plasma aliquots allowed a single freeze-thaw cycle prior to metabolomic processing for all specimens. All metabolomic data used for this analysis had been previously made available online (2), and untargeted discovery and targeted internal validation data had been obtained from baseline plasma specimens for all reported study participants. Glycerophospholipids were the most significantly dysregulated class of metabolites in our original untargeted discovery data. Discovery group data for this investigation resulted from 71 baseline subject specimens who underwent a targeted multiple reaction monitoring-stable isotope dilution-mass spectrometry (MRM-SID-MS) analysis using the Biocrates Absolute-IDQ P180 Kit (Biocrates Life Sciences, Innsbruck, Austria), which evaluates five classes of metabolites, including acylcarnitines (ACs), amino acids, hexoses, phospho- and sphingo-lipids, and biogenic amines, in an effort to reduce bias toward a particular class of metabolites. A subsequent internal validation study was completed on an additional 30 baseline subject specimens that underwent similar metabolomic analyses (Figure 1). These data were preprocessed, as previously described (2), prior to statistical consideration.

### Statistical Analysis

**Figure 2:**
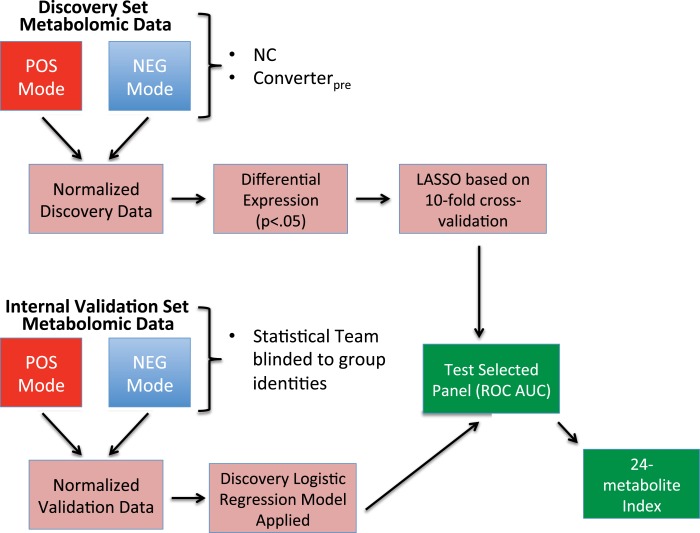
**Flow chart showing steps in biomarker model development**. Discovery cohort information was obtained from baseline specimen metabolomic data from subjects who remained cognitively normal (NC) throughout the study and baseline specimens from those that phenoconverted (Converter_pre_) during the study’s first 3 years. Discovery metabolomic data from positive and negative modes underwent normalization, followed by selection of significantly altered metabolites (*p* < 0.05), which were then annotated. The significant, annotated biomarker panel was then defined via a regularized learning method that features the LASSO restriction. The discovery biomarker panel selected is then tested using the receiver operating characteristic area under the curve (ROC AUC) method. With the statistical team blinded to group identities, the Internal Validation cohort data were similarly normalized and annotated. Internal Validation data were subjected to the results of the discovery logistic regression classifier and tested using the ROC AUC method. Combined data from the discovery and internal validation sets were used to develop a 24-metabolite index.

Statistical treatment of the data in this study was according to the same overall methods as described in our previous publication (2). The abundance measurements for metabolites (with a specific mass/charge ratio, *m*/*z*) in both positive and negative modes were expressed as intensity units that were initially normalized using log transformation and quantile normalization (Figure 2). For the 71 subjects in the discovery cohort, we calculated the level of differential expression for each metabolite using a *t*-test, comparing NC and Converter_pre_, constrained by *p*-value <0.05. Among these differentially expressed metabolites, we performed the feature selection using a regularized learning technique, which uses the least absolute shrinkage and selection operator (LASSO) penalty (7, 8). We first obtained the regularization path over a grid of values for the tuning parameter lambda (λ) through 10-fold cross-validation. The optimal value for λ obtained by the cross-validation procedure was used to fit the model. All the features with nonzero coefficients were deemed as biomarker candidates. This technique is known to reduce overfitting and variance in classification.

The classification performance of the selected metabolites was assessed using the ROC curve AUC. To maintain rigor of independent validation, the simple logistic model from the discovery set was fixed. The statistical team was blinded to the sample group identities of the internal validation cohort, which consisted of different NCs and Converter_pre_ subjects than those used in the discovery cohort. Any separation in values between NC and Converter_pre_ subjects for the final panel was evaluated using a robust method, the hidden logistic regression model with the maximum estimated likelihood (MEL) estimator (9). A combined classifier, based on the final biomarker panel for 101 subjects, within the discovery and internal validation groups, was developed to determine differences between NC and Converter_pre_ groups. The resulting combined classifier allowed the development of a plasma metabolite index (PMI), which provides a single predictive value of risk of phenoconversion in cognitively normal subjects observed over the 5-year interval. The PMI is obtained by mapping the log odds in a regularized logistic regression model on a 0–100 scale.

Positive and negative predictive value calculations used in this paper feature the direct measures of sensitivity and specificity defined from the ROC curves (10, 11) as well as the clinical prevalence from the literature (12), based on the disease in the specific population tested (13). Accuracy measures, which combine sensitivity and specificity for our biomarkers, were calculated for the 10-lipid and new metabolite panels. Accuracy values are calculated for potential cutoff probabilities of being diagnosed Converter_pre_ based on the ROC curve.

## Results

The clinical groups (see Table 1) were not significantly different (*p* ≥ 0.05) from each other based on gender, age, education, and *APOE* ϵ4 allele carrier percentages. *APOE* allele status was not a significant covariate, as previously reported (2). The ROC AUC with and without inclusion of *APOE* ϵ4 allele status in the classifier was not significantly different (*p* ≥ 0.05). Cognitive and phenoconversion details for the cohorts associated with this study are provided in Table 2. The memory *Z*-scores clearly decline from baseline to the post conversion (Converter_post_) state. Mean time to phenoconversion for all converters was 2.1 years. The discovery group had a mean time to phenoconversion of 1.5 years, while the internal validation group’s mean time to phenoconversion was 3.1 years. The mean time to phenoconversion was significantly longer for the internal validation group compared to the discovery group (Mann–Whitney *U Z*-score = −3.21, *p* = 0.0013).

**Table 2:** **Cognitive *Z*-scores and conversion diagnosis**.

	Cognitive *Z*-scores					Conversion Dx (aMCI/AD)	Years to phenoconversion [SEM]
	*Z*_att_ [SEM]	*Z*_exe_ [SEM]	*Z*_lan_ [SEM]	*Z*_mem_ [SEM]	*Z*_vis_ [SEM]		
Normal control (NC)							
Discovery	−0.17 [0.1]	−0.06 [0.1]	0.03 [0.1]	0.08 [0.1]	0.06 [0.1]	n.a.	n.a.
Internal validation	0.03 [0.1]	0.06 [0.2]	0.06 [0.1]	−0.05 [0.1]	0.27 [0.2]	n.a.	n.a.
Converter_pre_							
Discovery	−0.35 [0.2]	−0.54 [0.2]	−0.58 [0.3]	−0.81 [0.1]	−0.48 [0.3]	n.a.	n.a.
Internal validation	−0.42 [0.2]	−0.42 [0.4]	−0.03 [0.4]	−0.02 [0.1]	0.35 [0.3]	n.a.	n.a.
Converter_post_							
Discovery	−0.33 [0.2]	−0.60 [0.2]	−0.88 [0.3]	−1.7 [0.1]	−0.39 [0.3]	(16/2)	1.5 [0.5]
Internal validation	−0.31 [0.2]	−1.0 [0.4]	−0.75 [0.4]	−1.7 [0.1]	0.06 [0.3]	(5/5)	3.1* [1.2]

*Z_att_, attention composite *Z* score; *Z*_exe_, executive composite *Z* score; *Z*_lan_, language composite *Z* score; *Z*_mem_, memory composite *Z* score; *Z*_vis_, visuoperceptual composite *Z* score*.

*Conversion Dx represents the number of individuals who phenoconverted to the specific diagnosis: n.a., not applicable; aMCI, amnestic mild cognitive impairment; AD, Alzheimer’s disease. Note the prominent decline in *Z*_mem_ for the Converter subjects from Converter_pre_ to Converter_post_ consistent with phenoconversion to memory impairment. Also, note decline in other cognitive domains consistent with the diagnosis of AD in some subjects, which requires impairment in memory plus one other cognitive domain*.

*Mean time to phenoconversion was significantly longer for the internal validation converter group compared to the discovery converter group (Mann–Whitney *U Z*-score = −3.21, *p* < 0.01); SEM, standard error of the mean.

**Figure 3:**
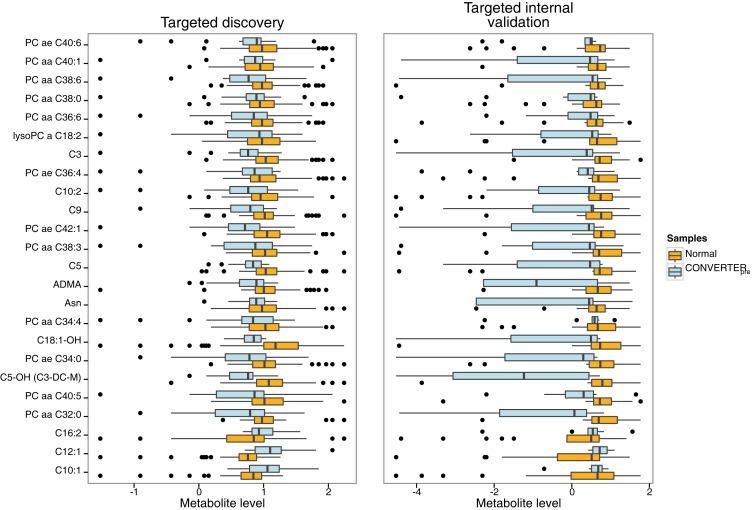
**Horizontal box and whisker plots of plasma 24 metabolite panel results for clinical groups in discovery and internal validation cohorts**. Comparative ranges of plasma metabolite levels for the targeted discovery and internal validation studies are displayed, allowing appraisal of metabolite results in the cognitively intact normal (orange) versus Converter_pre_ (light blue) groups. The box defines the interquartile range (IQR) with the vertical black line within the box representing the median value. The whiskers define the upper and lower 25% limits of the data, while the dots represent outliers (≥1.5 IQR lengths from the ends of the box). The normal group featured 53 subjects in the discovery and 20 in the internal validation cohorts, while the Converter_pre_ group included 18 and 10 subjects, for discovery and internal validation cohorts, respectively. Individual analytes are listed on the left vertical axis, while normalized metabolite levels are shown on the horizontal axis. All the Converter_pre_ analyte results are reduced in comparison to NC levels, except for three acylcarnitine species (bottom of figure), C16:2, C12:1, and C10:1, which are elevated. Note the higher variability of the internal validation set compared to the discovery set, due to less than half of the number of subjects in the former compared to the latter.

A total of 174 significant (*p* < 0.05) differentially expressed metabolites were defined in the discovery cohort. Of this group, 24 metabolites [13 glycerophosphatidylcholines (PCs), 9 ACs, 1 amino acid, and 1 biogenic amine] (Table 3) fulfilled the specific selection criteria established for the new biomarker panel. Three of the 24 metabolites, all belonging to the AC group (see bottom 3 entities in Table 3; Figure 3), had significantly increased levels, while quantities of the remaining metabolites were all significantly reduced in Converter_pre_ subjects compared to NC, for both discovery and internal validation groups (Table 3; Figure 3). Seven of the 24 metabolites were featured in our previously reported panel of 10 plasma lipids (2) (see top 7 in Table 3; Figure 3), and include a single AC (C3, proprionyl-l-carnitine), a single lysophosphatidylcholine (lysoPC a C18:2), and 5 PCs, with either ester (a) or ether (e) linkages (PC aa 36:6; PC aa 38:0; PC aa 38:6; PC aa 40:1; and PC ae 40:6). Nine novel ACs in the current panel include valeryl-l-carnitine (C5), hydroxyvaleryl-l-carnitine/methylmalonyl-l-carnitine (C5-OH/C3-DC-M), non-ayl-l-carnitine (C9), decenoyl-l-carnitine (C10:1), decadienyl-l-carnitine (C10:2)dodecenoyl-l-carnitine (C12:1), hexadecadienyl-l-carnitine (C16:2), and hydroxyoctadecenoyl-l-carnitine (C18:1-OH). This new panel also features asparagine (Asn), an amino acid, and asymmetric dimethylarginine (ADMA), a biogenic amine. All 7 novel PCs in this panel contain pairs of long chain fatty acids (FAs) (13–21 carbons), as did those in our previous report (2). The new PCs include PC aa C32:0, PC aa C34:0, PC aa C34:4, PC ae C36:4, PC aa C38:3, PC aa C40:5, and PC ae C42:1.

**Table 3:** **Components of the 24 metabolite biosignature for determining risk of phenoconversion from normal cognition to aMCI or AD**.

Metabolite name	Discovery cohort			Internal validation cohort	
	*p*-value	Log ratio (mean)	Log ratio (median)	Log ratio (mean)	Log ratio (median)
*PC ae C40:6*	0.0380	−0.3039	−0.0809	−0.2980	−0.2445
*PC aa C40:1*	0.0414	−0.2040	−0.0779	−1.1414	−0.1909
*PC aa C38:6*	0.0365	−0.3372	−0.2081	−0.9792	−0.1445
*PC aa C38:0*	0.0391	−0.2510	−0.0533	−0.5129	−0.1469
*PC aa C36:6*	0.0446	−0.3695	−0.1270	−0.2383	−0.1472
*lysoPC a C18:2*	0.0299	−0.3326	−0.0409	−0.3801	−0.1235
*C3*	0.0031	−0.4574	−0.2728	−1.2751	−0.3337
*PC ae C36:4*	0.0428	−0.3994	−0.0769	−0.6625	−0.2740
*C10:2*	0.0403	−0.4042	−0.1914	−0.1913	−0.2948
*C9*	0.0070	−0.4044	−0.2231	−0.8499	−0.2433
*PC ae C42:1*	0.0073	−0.4980	−0.3428	−1.3565	−0.3138
*PC aa C38:3*	0.0432	−0.4141	−0.1500	−0.8084	−0.2387
*C5*	0.0013	−0.2769	−0.1959	−0.6762	−0.2451
*ADMA*	0.0163	−0.2962	−0.1144	−1.0761	−1.5794
*Asn*	0.0441	−0.1788	−0.0891	−0.8982	−0.1933
*PC aa C34:4*	0.0346	−0.4353	−0.1906	−0.1430	−0.0835
*C18:1-OH*	0.0182	−0.2676	−0.3349	−1.2507	−0.2383
*PC ae C34:0*	0.0148	−0.4064	−0.2323	−1.2721	−0.4404
*C5-OH (C3-DC-M)*	0.0003	−0.4214	−0.3204	−2.1235	−2.0212
*PC aa C40:5*	0.0298	−0.4349	−0.1534	−0.5866	−0.4183
*PC aa C32:0*	0.0150	−0.4014	−0.1843	−1.4605	−0.6273
*C16:2*	0.0108	0.2767	0.0789	0.5001	0.0419
*C12:1*	0.0001	0.5075	0.3435	0.8748	0.2055
*C10:1*	0.0026	0.3642	0.2172	0.6121	0.0184

*In the metabolites listed, C_ species (e.g., C3) denote acylcarnitines (ACs). Phosphocholine (PC) metabolites display combined numbers of carbon atoms for their two acyl groups (sn_1_ and sn_2_ positions) (e.g., C38), whereas the combined number of double bonds (unsaturation) is displayed after the colon (e.g., C38:6). Acyl group linkages to choline backbone for PCs feature ester (a) or ether (e) linkage (e.g., PC ae C36:4). Asn, asparagine. ADMA, asymmetric dimethylarginine. LysoPC, lysophosphatidylcholine species, with only one acyl group, typically in the sn_1_ position. The discovery cohort provided significant differentially expressed metabolites between Converter_pre_ and NC. The column of *p* values indicates the significant differences for mean analyte values between the clinical groups for the discovery cohort. Log ratios represent the difference of the log-transformed values (mean or median) for Converter_pre_ and NC subjects. Negative log values indicate that levels (mean or median) of the analyte in Converter_pre_ < NC, while positive log values indicate that levels (mean or median) in Converter_pre_ > NC. NC, normal control subjects*.

**Figure 4:**
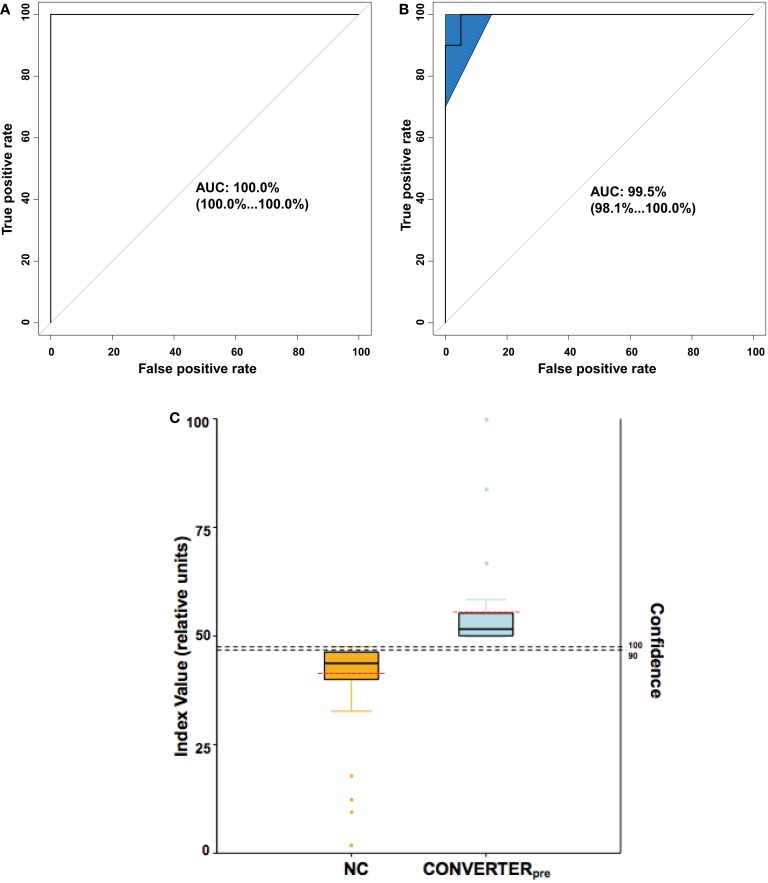
**Analytic representations to discriminate Converter_pre_ from normal control (NC) subjects**. **(A,B)** provide receiver operating characteristic (ROC) curves, whereas **(C)** depicts the calculated plasma 24 metabolite index (P24MI). **(A)** ROC area under the curve (AUC) for the Discovery cohort was equal to 1.00 with 95% confidence interval (in parentheses) ranging from 1.00 to 1.00. **(B)** ROC AUC for the internal validation cohort was 0.995, with 95% confidence interval (shaded area on plot) ranging from 0.981 to 1.00. **(C)** The P24MI results are depicted in vertical boxplots based on the logistic regression model that distinguishes between Converter_pre_ and NC groups. Solid black horizontal lines represent the mean value, while the dashed red horizontal lines represent the median value. Orange and light blue dots represent outliers (≥1.5 IQR lengths from the ends of the box). The higher index values (left vertical axis) are associated with an increased risk of phenoconversion to aMCI or AD, as seen in our Converter_pre_ subjects, with confidence (right vertical axis) of predicting phenoconversion transitioning from 90 to 100% at an relative index value of 48. Based on the calculated P24MI in our current dataset, a relative index value ≥49 represents a Converter_pre_ individual and a risk of phenoconversion of 100% within the 5-year study range. Note the relatively low variability of the P24MI for both the NC group and the Converter_pre_ group, with no overlap.

Receiver operating characteristic analyses (Figures 4A,B) of the plasma 24-metabolite panel yielded AUC measures of 1.00 and 0.995, for the discovery and internal validation groups, respectively. As a test on the accuracy of the 24-metabolite panel, a support vector machine (SVM) classifier was also developed on the discovery set and provided a similar ROC AUC (0.98) measure. Such precision allows the development of a plasma 24 metabolite index (P24MI) (Figure 4C) based on a regularized logistic regression model using the combined discovered and validated 24 metabolite values. The P24MI provides 100% confidence that subjects in our study with scores of ≥49 will phenoconvert to either aMCI or AD over the next 5 years.

Comparisons of our 10-lipid panel and expanded 24-metabolite panel are presented in Table 4, which define the comprehensive improvement provided by the expanded panel. Importantly, the presented PPV and NPV in Table 4 are derived using a conservative calculation method (10), and they feature similar published prevalence estimates of LOAD for female and male subjects aged 71–79 years: 2.33% for females, and 2.30% for males (12). Gender differences in LOAD prevalence grow significantly in subsequent decades, much higher in women, and is most likely due to their longer life expectancy (14).

**Table 4:** **Biomarker panel comparisons**.

Biomarker panel		Sensitivity	Specificity	ROC AUC	PPV/NPV (%)	Accuracy (%)
Gender	Cohort					
10 lipid (2)		0.9	0.85			85
Male					12.4/99.7	
Female					12.5/99.7	
	Discovery			0.96		
	Internal validation			0.92		
24 metabolite		1.00	0.95			95
Male					32.0/100	
Female					32.3/100	
	Discovery			1.00		
	Internal validation			0.995		

*ROC AUC, receiver operating characteristic area under the curve; PPV, positive predictive value; NPV, negative predictive value*.

## Discussion

We present an expanded plasma metabolite panel that attempts to maximize sensitivity, specificity, PPV, NPV, and accuracy in predicting risk of phenoconversion in a clinically asymptomatic cohort of seniors participating in a 5-year observational study. We included predictive assessments in the presentation of this 24-metabolite panel and in the retrospective analysis of our published 10-lipid panel (2) (see Table 4). It is important to note that our original lipidomic panel, while demonstrating the feasibility of risk identification using blood-based biomarkers for the preclinical stages of LOAD, was specifically defined to achieve approximately 90% sensitivity and specificity of classification utilizing the smallest number of analytes. These particular selection criteria were meant to provide interpretive simplicity and ease of implementation on a path toward a putative diagnostic assay. Since then, other investigators (15–17) have reported similar groups of phospholipids depleted in the blood of Alzheimer’s disease (AD) patients, while a recent nutritional intervention study provided an alternative validation of our initial lipidomic findings (18). Since the ultimate utility of a clinical diagnostic test will depend, at least in part, on a combination of safety, predictive accuracy, and cost, especially if used as a screening tool in asymptomatic subjects, we now provide a new metabolomic panel that maximizes predictive accuracy in the examined age group while maintaining safety and relatively low cost.

Despite the significant differences in time to phenoconversion between our discovery and internal validation groups (Table 2), we are encouraged that our metabolomic profile developed under a time to phenoconversion of 1.5 years also appears accurate up to 3 years prior to phenoconversion. We believe that analysis of serial specimens from our participants would provide extremely valuable information regarding analyte changes over time. Such analyses are yet to be finalized due to the associated expenses. However, we believe that insights on whether expansion of our metabolomic biomarker panel could be useful in raising predictive accuracy of phenoconversion risk would be independent of these serial analyses. While test sensitivity and specificity was improved, PPV and NPV, especially PPV remained limited by the low prevalence used for our age range (12). Importantly, our 24-metabolite panel provides an improved risk assessment regarding which subjects will develop aMCI or AD, and more importantly, which subjects will not.

The revised selection criteria for our 24-metabolite panel accounts for the inclusion of seven significantly dysregulated plasma lipid species from our original report (2), and 15 additional abnormal plasma lipids, a single amino acid, and a single biogenic amine. The 3 lipids included in our original 10-lipid panel but excluded from the current 24-metabolite panel were likely not considered due to more significant metabolites and the LASSO exclusion to avoid co-linearity. We assert that this novel plasma metabolite panel provides concordant, significantly altered analytes based on the specific selection criteria and statistical stringency used. These plasma biosignatures of phenoconversion risk primarily feature dysregulated lipid species, with the majority being reduced in plasma compared to normal. The significant reduction in both PC and AC species in peripheral blood supports the hypothesis put forth by our group (2) and others (5, 15, 18–20) that abnormalities in lipid networks may not only represent biomarkers for, but may be integral to the development of specific neurodegenerative pathologies, including LOAD. Similarities in the dysregulated lipid networks within brains from human LOAD and transgenic mouse models of early-onset AD (EOAD) suggest disruptions in the levels of certain bioactive lipids, including glycerophospholipids, ceramides, and sphingomyelins, highlighting the utility of lipidomics for investigating these conditions (21). Future assessments may elucidate shared as well as distinct etiologic mechanisms in both EOAD and LOAD and dictate particular therapeutic options to target the differences in their pathobiologic networks.

**Figure 5:**
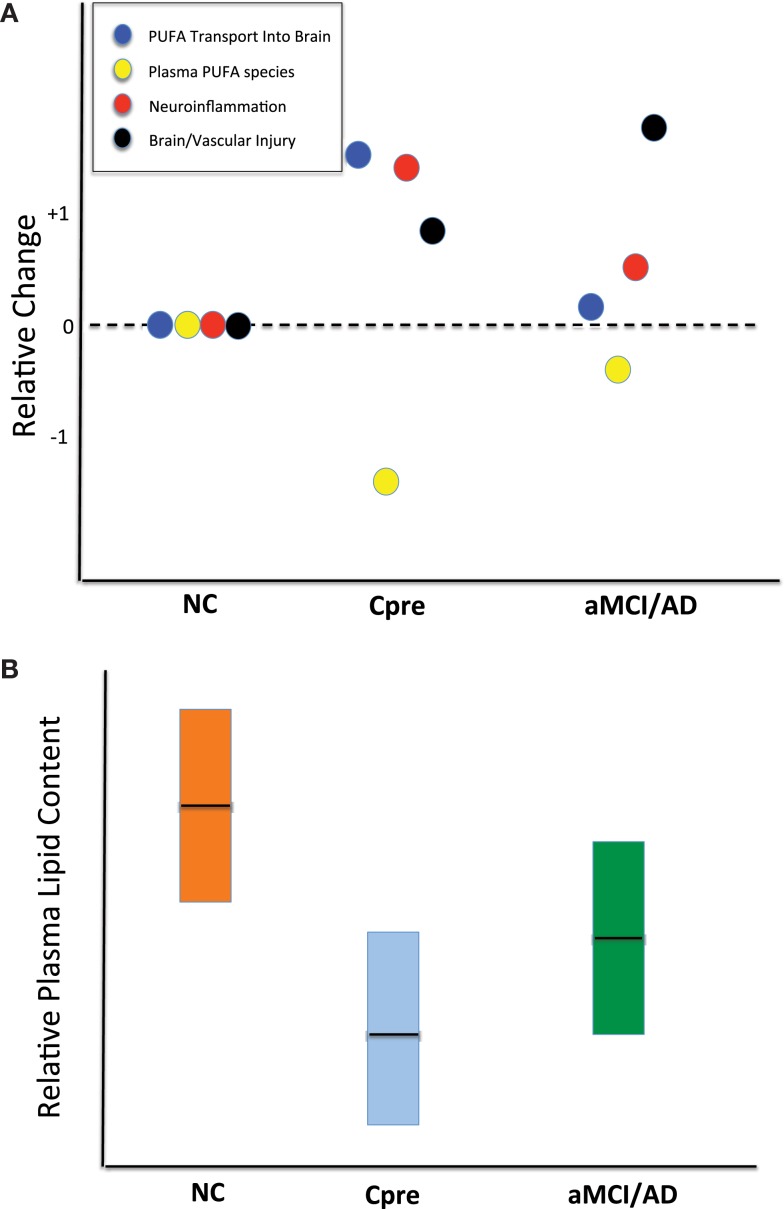
**Schematic representations of potential alterations within brain and peripheral blood responsible for reduced plasma phospholipid levels**. **(A)** Qualitative dot plot of differential changes occurring in cognitively normal control (NC), cognitively normal Converter_pre_ (C_pre_) subjects, and those with amnestic mild cognitive impairment or Alzheimer’s disease (aMCI/AD). Note that all four processes (represented within boxed legend) are at the zero relative level in the NC subjects. The C_pre_ subjects could show significant polyunsaturated fatty acid (PUFA) transport into brain to replenish lost substrate as a result of neuroinflammation or other brain injury. The increase in PUFA flux into the brain is an attempt to compensate for ongoing injury and results in a marked reduction in the plasma levels of molecules carrying those lipid species. Dark horizontal line within boxes represents proposed mean. **(B)** Qualitative plasma phospholipid biomarker results, previously quantified (2), which may be better interpreted via the theory proposed in **(A)**. Dark horizontal line within boxes represents proposed mean. The full explanation for this metabolic phenomenon in the C_pre_ subjects remains to be elucidated.

In our previous (2) as well as our current plasma biomarker panel, all the PCs are notably reduced during the preclinical stages of LOAD. Similar PC reductions have been documented in AD brains (22) and attributed to pathologic activation of phospholipase A_2_ (PLA_2_) (22, 23). Using current analytic methods, dysregulated lipid metabolism has been confirmed, with reductions in specific PCs noted in brain (24), plasma (5), and serum (25) of AD subjects compared to controls. With PLA_2_ activity known to form lyso PCs, lack of significant central elevations in this phospholipid byproduct may relate to their rapid re-acylation to form PCs for repair (or attempted repair) of membranes (26) or to generation of downstream metabolites. The mechanistic link between reduction of brain lipid in association with LOAD, and in peripheral blood, has yet to be fully elucidated. Interestingly, the PCs in our 24-metabolite panel all feature polyunsaturated fatty acids (PUFAs) (Table 3), as has been reported by others (25, 27). While the brain has the capacity to generate all the lipid species it requires for normal function, along with most saturated and monounsaturated FAs (28, 29), specific substrates required to maintain brain lipid homeostasis, especially sources of energy and certain PUFAs, are delivered to the brain via the bloodstream. In normal brain metabolic processing, phospholipid components are efficiently recycled and have relatively long central half-lives (28). Essential PUFAs such as docosahaxaenoic acid (DHA; 22:6 n-3) and arachidonic acid (AA; 20:4 n-6) provide structural functionality as phospholipid components in bilayer membranes. Once released, either directly or through byproducts, they are known to participate in signal transduction processes that have positive and negative consequences within cells (30). Under conditions where brain membrane lipids undergo catabolism (e.g., oxidative stress, or neuroinflammation), the downstream intermediates often are not recycled to the membrane and thereby increase the demand for lipid precursors from the bloodstream (Figure 5). Such precursors exist in plasma as unesterified FAs (≤22 carbons) bound to albumin, or as esterified FA species (>22 carbons) within phospholipids preferentially transported within circulating lipoproteins (30). Esterified FAs can be converted to unesterified forms via lipases within the lipoproteins or circulating within the blood (31–33). Flux of unesterified FAs into the brain, across the blood–brain barrier (BBB), is rapid and occurs via simple diffusion and possibly via facilitated transport (30). All unesterified FAs entering the brain are immediately esterified by acetyl-CoA-synthase (34–37), preventing their diffusion back to blood and preparing them for incorporation into lipid biosynthetic pathways. Activation of phospholipases (e.g., PLA_2_) with increased oxidative stress is implicated in diminishing PUFAs from membrane lipids (16). We have observed elevated levels of oxidative lipid metabolites in our at-risk preclinical subjects, that do not reach statistical significance, but reach significant elevation in plasma from symptomatic LOAD subjects compared to controls [unpublished data]^1^. Hartmann and colleagues (18) provided an indirect test to this hypothesis of depleted substrates (Figure 5A), in their investigation of a medically regulated nutritional supplement (Souvenaid^®^) in a randomized, placebo-controlled, double-blind clinical trial in subjects having mild dementia, attempting to stimulate *de novo* PC synthesis via the Kennedy pathway (38). They report significant elevations in five specific blood-derived PCs with the supplemental agents, including members of our original plasma 10-lipid panel. Restoration of blood lipids with dietary supplements has been proposed as beneficial in both preclinical and mild AD by stabilizing synaptic membrane function (39), and network connectivity (40). In another recent publication (41), the authors propose that redox reactive autoantibodies are produced in CSF and blood as a result of exposure to oxidizing agents (in prodromal or manifest AD). Moreover, they proposed utilizing the autoantibody levels as disease biomarkers to differentiate control subjects from those with MCI or AD. From our perspective, such phospholipid autoantibodies are poised to preferentially bind to specific plasma phospholipids, with resultant clearance of the conjugates from blood plasma, making brain lipid substrates less available for entry into the brain. Recent data indicate a role for ACs beyond β-oxidation (42), including neuroprotection by increasing antioxidant activity, modulating membrane composition, assisting with lipid biosynthesis, participating in gene regulation, enhancing cholinergic neurotransmission, and improving mitochondrial function. Altered AC levels in those destined to phenoconvert to AD, therefore, may parallel central alterations in neuroprotection and/or bioenergetic capacity.

Finally, the reduced abundance of asparagine (asp) and asymmetric dimethylarginine (ADMA), in Converter_pre_ compared to NC, provide insights into orthogonal dysregulated networks in the preclinical stages of LOAD. CSF and plasma asparagine levels are known to be reduced in AD subjects compared to controls (43). Depletion of asparagine from the CNS has been associated with reversible altered mental status in children and adults, including short-term memory impairment in the elderly (44). Asparagine’s primary role is within proteins (45), affording a common site for N-glycosylation and providing unique structural characteristics at the ends of α-helices and within β-sheets (46). The brain, compared to peripheral organs, is particularly dependent on intrinsic production of asparagine due to limited transport across the BBB (47). ADMA is produced from a post-translational modification of polypeptides by specific methyltransferases, with subsequent release into plasma following cellular protein turnover (48). Elevated ADMA levels in blood have been consistently associated with cardiovascular disease (CVD) risk factors, such as hypertension or hypercholesterolemia (48, 49). Within blood and other tissues, ADMA is considered the primary inhibitor of nitric oxide synthase (NOS), and thereby, a regulator of nitric oxide (NO) production. Brain-specific NOS (nNOS or NOS1) (50), and consequently NO production, is regulated by the relative concentrations of substrate, arginine, versus ADMA (48). Although increased NO concentrations have been associated with neuronal cell death, NO has been implicated in important synaptic actions, including learning and memory (51). While ADMA levels in CSF and blood of AD patients have not provided consistent findings (52), we have not found previous reports of ADMA plasma levels in preclinical AD subjects. The reduced ADMA levels in our preclinical AD (Converter_pre_) subjects, and thereby the implied elevation of NO vasodilator activity compared to matched controls, may indicate a compensatory process, possibly triggered by the presence of oxidative stress that increases NO production in the early stages of AD (53). Additional investigations are required to further elucidate these associations.

The potential for a highly accurate early screening test for AD raises important questions about the value of such testing, especially given that AD is a condition for which no cure exists and treatment options are extremely limited. In other contexts, the potential disutility associated with receiving bad health news (54, 55) and with discrimination based on test results, particularly in an employment or insurance context, has been recognized (56). On the other hand, information from early screening may produce utility by reducing uncertainty about the future and allowing individuals to optimize key economic decisions related to consumption, retirement, and future planning (57, 58). In addition, because significant limitations and rapid declines in financial capacity are a hallmark of patients with early stage AD (59–62), earlier diagnosis may also yield value in the form of averted financial losses. Individuals with AD that is too early to diagnose may be susceptible to financial exploitation and may have trouble managing day-to-day household financial responsibilities such as paying bills on time. Accurate LOAD testing may help families better recognize and respond to those financial decision-making deficits – such as by changing the financial head of household or instituting other checks and balances (58) – before major financial problems occur. The scale and scope of negative financial outcomes associated with AD in the prediagnosis period may be substantial but as yet remain unquantified. Finally, the advent of a predictive LOAD diagnostic is likely to advance researchers’ ability to develop and test novel AD therapeutics. Responding to the projected future financial burden imposed by LOAD, and the potential sources of value from predictive testing, many states have prioritized early detection in their future preparations for AD (63).

As highlighted in the most recent report of the Presidential Commission for the Study of Bioethical Issues (64), ethical reflection and review need to be integrated into the research process from the planning phase to produce treatments and therapies that best meet the patient’s values and goals. Procedures should be implemented to ensure patient and public participation in the design of ethical research protocols, development of diagnostics and treatments, and a delivery process for predictive AD diagnostics. To achieve these goals, accurate and transparent public communication is needed, along with an emphasis on pre- and post-test counseling, as underscored in recent guidelines for AD testing (65).

We acknowledge the residual limitations provided by the relatively small, homogenous cohort of subjects used in this investigation, which is a subset of our previously reported study participants (2). We believe that there are advantages to a longitudinal study design that cannot be replicated in larger cross-sectional or case–control studies, however, especially for defining and directly investigating the preclinical state. Despite the added cost and time required, longitudinal observational studies allow the direct determination of the included preclinical period and to time the transition to clinical disease quite accurately. It is through analyses of preclinical biospecimens directly determined through such observations that specific, temporally related disease mechanisms can be accurately determined. As a result of longitudinal clinical and limited correlative biomarker determinations, we have helped define potential preclinical dysregulated plasma lipids and other metabolites within our study group. Similar preclinical mechanisms can only be inferred using methods that compare health to disease. We remain committed to a full analysis of all of our longitudinal specimens obtained from our Rochester/Orange County Aging Study subjects. In the meantime, however, an external validation study is underway in which we are evaluating plasma specimens from a larger, more ethnically diverse, and slightly younger subject cohort to discern the applicability of our current metabolomic biomarker panels beyond our strictly defined cohort (2). External validation of our findings remains a critical component that currently limits the impact and utility of our results.

We also acknowledge the possibility of overfitting of the classifier model to our limited set of subjects in this investigation, despite our attempt to minimize such effects with the statistical methods used. We present the current findings as a starting point, therefore, for the external validation studies that are currently underway. Optimal external validation of our biomarker panels will require plasma samples that are obtained in similar subjects, under comparable rigorous collection and processing procedures. In our case, specific details to be followed would include morning blood collections in a limited time window, following an overnight fast and withholding morning medications, and minimizing plasma freeze-thaw cycles prior to metabolomic analysis. It seems unlikely that currently available specimens from external cohorts will meet such strict criteria, but application of our biomarker panels to such disparate specimens will instruct us regarding what similarities, if any, may exist related to preclinical disease biosignatures despite different demographics and sample collection/processing methods.

The alteration in specific analyte species during the preclinical stages of LOAD from our studies is consistent with results from other groups (25, 27, 38) and provides evidence for unique metabolomic dysregulation, especially related to plasma lipids, during the preclinical and clinical LOAD stages. While the theoretical basis for the significant preclinical lipid reductions within plasma during preclinical LOAD remains unconfirmed, there are several mechanistic reasons for their occurrence that can be readily tested. We encourage other investigators to advance our understanding of such postulates through independent validation studies. The dysregulated analyte species found in our study subjects appear to suggest at least several altered metabolic networks, distinct from amyloid and tau, during the preclinical LOAD stages, which if supported by additional investigations, may encourage development of new potentially disease-modifying interventions. The current shift toward diagnostics that help define preclinical LOAD (i.e., biomarkers from blood, CSF, and neuroimaging) is expected to stimulate a new class of secondary prevention clinical trials that feature novel or repurposed therapeutics. Enrichment of asymptomatic at-risk individuals for participation in such trials would depend on using accurate, safe, and inexpensive subject selection methods. The optimal biomarker method(s) could also allow serial monitoring of specific pathobiologic networks that could herald therapeutic failure (or success). Such biomarker approaches may not only allow improved patient safety but also mitigate overall study costs. Novel capabilities provided by preclinical biomarkers, we believe, will help stimulate resurgence in therapeutic development for LOAD by the biopharmaceutical industry. We remain encouraged that through a heightened awareness of all stakeholders in our society regarding the possible utility of preclinical biomarkers, through education and dialog, we may be better positioned to cope with and eventually overcome the devastating effects of LOAD on the world’s population.

## Authors Contribution

MF, AC, MM, and HF conceived this investigation. MM was primarily responsible for coordinating the recruitment of patient samples and clinical data collection. AC was primarily responsible for the metabolomic analyses. XZ, MT, and MN provided statistical analyses of the metabolomic data and classifier development. MF, XZ, AC, MT, MN, AS, MM, and HF participated in data interpretation. MF, XZ, and MM produced all tables and figures. MF, SZ, AC, MO, SC, and MM performed the literature search. CG provided neuro-economics perspectives. KF provided the neuro-ethics perspectives. All the authors participated in writing the manuscript and editing for content. Final manuscript preparation and edits were performed by MF and HF.

## Conflict of Interest Statement

The authors declare a potential conflict of interest and state as follows. Drs. Massimo S. Fiandaca, Xiaogang Zhong, Amrita K. Cheema, Mark Mapstone, and Howard J. Federoff have patents filed on their behalf through Georgetown University. Dr. Massimo S. Fiandaca is named as a co-inventor on a provisional patent application filed by Georgetown University and the University of Rochester related to the specific biomarker technology described in this manuscript. Dr. Xiaogang Zhong is named as a co-inventor on a provisional patent application filed by Georgetown University and the University of Rochester related to the specific biomarker technology described in this manuscript. Dr. Amrita K. Cheema is named as a co-inventor on a provisional patent application filed by Georgetown University and the University of Rochester related to the specific biomarker technology described in this manuscript. Dr. Michael H. Orquiza has no disclosures. Ms. Swathi Chidambaram has no disclosures. Dr. Ming T. Tan has no disclosures. Dr. Carole Roan Gresenz has no disclosures. Dr. Kevin T. FitzGerald has no disclosures. Dr. Mike A. Nalls has no disclosures. Dr. Andrew B. Singleton has no disclosures. Dr. Mark Mapstone is named as a co-inventor on a provisional patent application filed by Georgetown University and the University of Rochester related to the specific biomarker technology described in this manuscript. Dr. Howard J. Federoff is named as a co-inventor on a provisional patent application filed by Georgetown University and the University of Rochester related to the specific biomarker technology described in this manuscript.

## Footnotes

^1^Cheema AK, Personal communication (2015)
